# Honesty is the best policy: A brief overview of retraction notices.

**DOI:** 10.3126/nje.v6i4.17253

**Published:** 2016-12-31

**Authors:** Ritesh G Menezes, Pradhum Ram, Huda Fatima, Brijesh Sathian

**Affiliations:** 1 College of Medicine, University of Dammam, Dammam, Kingdom of Saudi Arabia; 2 Albert Einstein Medical Centre, Philadelphia, PA, United States of America; 3 Dow Medical College, Dow University of Health Sciences, Karachi,Pakistan; 4 Manipal College of Medical Sciences, Pokhara, Nepal

Authenticity is the foundation of science. Without our individual involvement, participation, interaction, sincerity and work ethic, our collective medicine would cease to exist. So also is the case of the advancement of medicine. One of the main means to progress, in this field is the conduct of research, which would eventually have an impact on the world we live in.

The conduct of research requires one too many qualities in both the individual and the team, from the inception, the planning, and the execution until the final publication. It is a hard fought journey with many daunting challenges. And because of the humongous power that rests on the author's shoulders, a great amount of responsibility tags along with it given that at each and every step a potential for human or other error does exist and a dedicated and sincere effort on the part of the individual and the team must be taken to prevent this from happening. This responsibility is nothing but conducting honest research bearing in mind the implications of falsehood. And this is where the concept of retraction notices comes into the picture.

Back in the 1970s when publications were not as abundant, retraction notices were an inexact undertaking and posed quite a challenge. There were no strict guidelines and each journal had its own format to retract an article, none being better than the other. The threat of legal proceedings against the editors or journal publishers loomed large [  1 ]. The problems were compounded as some journals did not employ a policy of retraction at all while others, employed methods such as a page at the end of the issue, that wasn't indexed in the contents. Some others, such as journals that published review articles, primarily did not believe in the need for retractions at all while others hid the notices between advertisements or boxed these notices at the bottom of pages [  1  , 2 ]. Despite these methods having little precedent with regards to style, they garnered importance through the 80s and by the early 90s moving into the 21stcentury, concrete guidelines were beginning to form. Numerous studies have been conducted to standardize this process of issuing a retraction and although obvious flaws such as issuing a retraction without citing a cause for it to name a few still exist, the overall process has made tremendous progress [  3 ]. International committees such as the Committee on Publication Ethics (COPE) have since formed and the process of retraction is now setting up to have standard guidelines to follow and recent studies have shown that within the last decade the presence of retraction policies within journals have doubled which shows promise. Further, many journals have cited a standard set of guidelines while forming their own retraction policy [  1 - 3 ].

A retraction notice is issued in the journal where the said article is published by a particular author or team of authors, if there is evidence of misconduct such as plagiarism, data falsification or fabrication that could seriously undermine the reliability of the article itself. From the standpoint of the author, an honest error might have unknowingly resulted in the apparent misconduct. Therefore, distinguishing human error from actual misconduct is of utmost importance for the editors of the journal [  3 ]. The editors of the journal then write a letter usually to the corresponding author of the article asking for a logical explanation for the conundrum in an effort to screen for human error and to avoid unnecessary injustice. If the author or the team is found guilty of unethical research and publication practices, then a motion is passed to have the article retracted and it is considered null and void for all practical purposes.

Now, there are a few more things that are important here. Firstly, when an article is under review at any journal, multiple checks are employed to scout for plagiarism and these methods are more stringent with increasing impact factor of the journal [  1 ]. However, instances where falsification or fabrication of data is implied pose more of a challenge given the tremendous window for manipulation of data. The task of proving ethical misconduct remains difficult in such cases. However, once proven, the journal issues the retraction notice and it becomes important from the standpoint of educational ethics to conduct a post-publication review of other articles published by the same author in order to check authenticity. In articles involving multiple participants or investigators identifying individual participants whose work may have led to a falsified result remains a challenge given the multitude of roles played by every participant. 

 The classic example that is mentioned about such retraction articles is one of Eric T. Poehlman. He was an internationally recognized scientist, but his colleagues were convinced that he had falsified data and thus a full scale investigation was launched into his works following which he pleaded guilty. However, for years prior articles published by him were referred to and he was considered an authority in the field of obesity and ageing [  4 ]. 

 Imagine using the wrong treatment protocols, an unnecessary investigation modality or the wrong drug to treat patients based on unethical research. Therefore, it is imperative for us as a scientific body to realize the importance of not only being aware and reporting such articles but also ensuring that it never occurs in the first place.

It has been said that practice makes progress and progress leads to perfection, and we have made immense progress by the formation and revision of retraction policies and this has understandably led to the publication of more authentic literature in recent times. However, multiple hurdles still do exist and by virtue of authentic studies we hope to overcome them with the eventual goal of a world of publication free of misconduct.
